# A Recurrent *STAT5B^N642H^* Driver Mutation in Feline Alimentary T Cell Lymphoma

**DOI:** 10.3390/cancers13205238

**Published:** 2021-10-19

**Authors:** Matthias Kieslinger, Alexander Swoboda, Nina Kramer, Patricia Freund, Barbara Pratscher, Heidi A. Neubauer, Ralf Steinborn, Birgitt Wolfesberger, Andrea Fuchs-Baumgartinger, Richard Moriggl, Iwan A. Burgener

**Affiliations:** 1 Department for Companion Animals and Horses, Division of Small Animal Internal Medicine, University of Veterinary Medicine Vienna, 1210 Vienna, Austria; alexander.swoboda@yahoo.de (A.S.); nina.kramer@vetmeduni.ac.at (N.K.); patricia.freund@vetmeduni.ac.at (P.F.); barbara.pratscher@vetmeduni.ac.at (B.P.); birgitt.wolfesberger@vetmeduni.ac.at (B.W.); iwan.burgener@vetmeduni.ac.at (I.A.B.); 2 Department of Biomedical Sciences, Institute of Animal Breeding and Genetics, University of Veterinary Medicine Vienna, 1210 Vienna, Austria; heidi.neubauer@vetmeduni.ac.at (H.A.N.); richard.moriggl@vetmeduni.ac.at (R.M.); 3 Genomics Core Facility, VetCore, University of Veterinary Medicine Vienna, 1210 Vienna, Austria; ralf.steinborn@vetmeduni.ac.at; 4 Department of Pathobiology, Institute of Pathology, University of Veterinary Medicine Vienna, 1210 Vienna, Austria; andrea.fuchs@vetmeduni.ac.at

**Keywords:** STAT5B, driver mutation, feline alimentary lymphoma, STAT3

## Abstract

**Simple Summary:**

Human gastrointestinal lymphomas are rare diseases with an incidence of one per 1,000,000 inhabitants per year. This paucity poses a major challenge in unravelling their underlying mechanism. In comparison, lymphoma is the most common malignancy in domestic cats and the gastrointestinal (GI) tract is the most common location for this disease. Here, we identify the driver mutation *STAT5B^N642H^* in feline alimentary lymphoma, thereby establishing felines as a potential new model for a rare and incurable human T cell disease.

**Abstract:**

Alimentary lymphomas arising from T cells are rare and aggressive malignancies in humans. In comparison, they represent the most common anatomical form of lymphoma in cats. Due to the low prevalence in humans, the underlying pathomechanism for these diseases is poorly characterised, limiting experimental analysis and therapeutic exploration. To date, activating mutations of the JAK/STAT core cancer pathway and particularly the STAT5B oncoprotein have been identified in human enteropathy-associated T cell lymphoma. Here, we describe a high homology of human and feline STAT3 and STAT5B proteins and strong conservation at the genomic level. Analysis of 42 samples of feline T cell alimentary lymphoma reveals broad activation of STAT3 and STAT5B. Screening for known activating mutations in *STAT3* or *STAT5B* identifies the presence of the *STAT5B^N642H^* driver mutation in feline enteropathy-associated T cell lymphoma in 7 out of 42 (16.67%) samples in total. Regarding lymphoma subtypes, the majority of mutations with 5 out of 17 (29.41%) cases were found in feline enteropathy-associated lymphoma type II (EATL II). This identification of an oncogenic *STAT5B* driver mutation in felines recapitulates the genetic situation in the corresponding human disease, thereby establishing the cat as a potential new model for a rare and incurable human T cell disease.

## 1. Introduction

Lymphoma is the most common malignancy in domestic cats, and the gastrointestinal (GI) tract is the most common location for this disease [1,2]. Alimentary (gastrointestinal) lymphoma is characterised by infiltration of the upper or lower GI tract with neoplastic B- or T-lymphocytes with or without mesenteric lymph node or hepatosplenic involvement [3]. Alimentary T cell lymphomas arise in the mucosal-associated lymphoid tissue, which consists of lamina propria lymphocytes and intraepithelial lymphocytes representing largely T cells [4]. These T cell lymphomas can be subdivided histopathologically into low grade (small cell, well-differentiated) or intermediate and high grade (large cell, less differentiated) types, with the clinical features of intermediate and high grade being similar in biological properties [3]. The most common subtype is low grade and is known as enteropathy-associated T cell lymphoma (EATL) type II, or small cell lymphoma, accounting for 10–20% of all tumours with incidences ranging from 41 to 200 cases per 100,000 cats reported each year [5]. Although EATL type I, or large cell lymphoma, is less common, the prognosis is significantly worse. The standard of care for EATL type II can extend life for two years or more, while care for type I rarely extends life more than 6–9 months [6].

Human primary gastrointestinal lymphomas are very rare, accounting for less than 5% of all non-Hodgkin lymphomas (NHL) [7,8]. Feline EATL type I most closely resembles human enteropathy-associated T cell lymphoma (EATL; previously designated EATL type I) [9]. Feline EATL type II shows parallels to human monomorphic epitheliotropic intestinal T cell lymphoma (MEITL; previously designated EATL type II) as well as indolent digestive T cell lymphoproliferative disease (ITCL) [9,10,11]. Furthermore in the WHO classification for human and veterinary medicine all nodal and extranodal T cell lymphomas which do not correspond to a defined entity of T cell lymphoma are classified as peripheral T cell lymphoma not otherwise specified (PTCL-NOS) [12]. Prognosis of EATL and MEITL is considered poor with a five-year overall survival of 11–20% and no standardised treatment protocols, whereas ITCL progresses more slowly with some cases developing into aggressive T cell lymphoma [13,14,15]. The pathogenesis of human EATL and MEITL is poorly understood and the rarity of the disease in humans is the major factor limiting the identification of putative oncogenic events and new targeted treatment strategies. In contrast, feline alimentary lymphoma is more common, making it a potentially suitable animal model for a comparative understanding of the human condition [11]. Elucidating pathogenic mechanisms underlying human intestinal lymphoma is highly needed. By exploring the pathogenetic mechanisms involved in feline intestinal lymphoma, it should be possible to improve our understanding of the human disease.

The Janus kinase (JAK)/signal transducer and activator of transcription (STAT) pathway is an evolutionarily conserved signaling pathway, mainly regulating components of cell division, apoptosis, metabolism, chromatin regulatory processes or cell fate decisions [16,17]. STAT proteins are highly expressed and hyper-activated by tyrosine and serine phosphorylation in over 70% of all human cancer types, thereby constituting a critical node in the signaling network of tumour cells [18,19]. Particularly STAT3 and STAT5B are constitutively activated either by gain-of-function mutations in JAKs or other upstream oncogenes or less frequently by activating mutations and have been linked to tumour initiation and progression [20,21]. There is often synergy between STAT3/5 signaling in cancers cells, which culminates in overlapping target gene spectra such as D type cyclins, *c-myc* or *bcl-2* family members, best exemplified by the mutational landscape of recurrent somatic STAT3 and STAT5B mutations in peripheral T cell lymphoma patients [22]. Until recently, the genetic basis of human gastrointestinal lymphoma was poorly characterised, and was mainly restricted to copy number alterations [23,24]. Targeted whole-exome and transcriptome sequencing-based analyses have begun to reveal the spectrum and frequency of genetic abnormalities in human intestinal lymphoma [25,26,27,28,29]. Today we know from sequencing the cancer genome that the JAK/STAT pathway is commonly mutated in human EATL, MEITL and ITCL. Initial studies, focused on characterising the mutational profile of MEITL, revealed frequent mutations in *STAT5B^N642H^* (43% of patients), occurring within the SH2 domain and leading to a constitutively active protein [25]. Additionally, *STAT3^Y640F^* and *STAT3^D661Y^*, activating mutations located in the SH2 domain have been observed, albeit at a lower frequency in EATL/MEITL (7% of patients) [25,26,27,28]. Interestingly, *STAT3* and *STAT5B* mutations were found to be mutually exclusive, but the dominant transcription factor for neonatal programming of γδ T cells is STAT5, suggesting a key role for its activation, even in the absence of somatic gain of function mutations [30].

Here, we examine the possibility that the overlap in feline and human alimentary lymphoma is not restricted to clinical signs, gastrointestinal location, histology and immunophenotype, but also extends to the genetic level [11]. Since alimentary lymphoma is the most frequent neoplasm in cats, defining common pathogenic mechanisms with the corresponding human disease offers the potential to improve understanding of this rare human disease, identify diagnostic and prognostic markers and develop therapeutic regimens.

## 2. Materials and Methods

### 2.1. Biological Samples

The samples used in this study were collected retrospectively from formalin-fixed paraffin-embedded (FFPE) tissue blocks generated during diagnostics of feline lymphoma at the University of Veterinary Medicine, Vienna between 2008 and 2020. Available samples with a primary diagnosis of T cell lymphoma, EATL I, EATL II or PTCL-NOS located in the intestine or the mesenteric lymph node were included in the study (*n* = 42). Informed consent of the patient owner was obtained in all cases, following the guidelines of the local Ethics and Animal Welfare Committee. Cases had been classified histopathologically and immunohistochemically by a veterinary pathologist according to the World Health Organization classification [31]. Briefly, EATL type I was diagnosed in case of transmural infiltration with lymphoid cells characterised by a large round or irregularly folded nucleus. EATL type II in case of mucosal lymphoma of cells with a small round and dense nucleus showing epitheliotropism. A diagnosis of PTCL-NOS was made in cases of exclusive infiltration of the mesenteric lymph node or infiltration of the intestinal muscularis or subserosa without the involvement of the mucosa (see also Figure 2A). Mitotic count (Table S1) was assessed by selecting a cellular region at the periphery of the tumour and counting mitotic figures in ten consecutive high-power fields (40× objective, 10× ocular FN 22 mm) [32]. Clinicopathologic data is provided in Table S1.

### 2.2. Immunohistochemistry

For immunohistochemical staining (IHC), heat-induced epitope retrieval was performed in citrate buffer at pH 6.1 (Dako/Agilent Technologies, Santa Clara, CA, USA, S2031). IHC was performed for STAT3 (BD, Heidelberg, Germany, 610189, 1:200) and STAT5B (Santa Cruz sc1656, 1:200) using the UltraVision LP Detection System Kit (Thermo Scientific, TL-060-NL, Schwerte, Germany) according to the standard protocol.

IHC sections were scanned using a Leica DMi8 microscope with Leica Application Suite X software v3.6 (Leica Microsystems, Wetzlar, Germany). For the quantification of STAT3 and STAT5B staining, marker signal was quantified on the cellular level using the “Nuclear Segmentation” algorithm of the HistoQuest software v6.0 (TissueGnostics GmbH, Vienna, Austria). An area of at least nine high-power fields (×200) was analysed per sample. Cells were identified based on nuclear haematoxylin staining. Total AEC chromogen staining was measured per cell and per nucleus. Cells with an AEC positive area of at least 6 µm^2^ were considered stained in all groups. One sample of the EATL II group was excluded from evaluation due to insufficient quality.

### 2.3. DNA Isolation

Genomic DNA was isolated from FFPE tissue sections using the ReliaPrep^TM^ FFPE gDNA Miniprep System Kit (Promega, Madison, WI, USA) according to the instructions of the manufacturer. DNA purity (A_260/280_ ratios: ≥1.8 to 2.1) and concentration were measured using NanoDrop^TM^ One^C^ (Thermo Scientific, Waltham, MA, USA).

### 2.4. PCR Screening

Primers spanning the feline *STAT5B c.1924A>C* (p.N642H), *STAT3 c.1919A>T* (p.Y640F) and *c.1981G>T* (p.D661Y) potential mutation sites in the SH2 domain of the respective genes to search for equivalent mutations in feline T cell lymphomas were designed (Table S2). PCR amplicons were run on a 2% agarose gel. Respective bands were isolated and purified using a GeneJET Gel Extraction Kit (Thermo Scientific, K0691) according to the standard protocol. Mutational hotspots for *STAT3* and *STAT5B* were analysed by Sanger sequencing (Microsynth AG, Balgach, Switzerland).

Two sets of primers determined the FeLV status. One previously published set spans a highly conserved part of the unique region (*U3*) of the long terminal repeat (LTR) among exogenous FeLV-A, -B and -C isolates [33]. The second FeLV primer set detects a sequence in the *U3* region that does not discriminate between exogenous virus DNA and endogenous proviral elements in the feline DNA (Table S2).

PCR was performed according to the standard protocol using the Phusion™ Hot Start II High-Fidelity DNA Polymerase Kit (Thermo Scientific, F549S). In brief, a 20 µL reaction was mixed using Buffer HF according to a standard 3-step protocol. PCR thermal cycling protocol: 30 s 98 °C, 35–40× (30 s 98 °C, 30 s 60 °C, 1 min 72 °C), 5 min 72 °C, hold at 4 °C. Primers were designed in Primer3 (release 4.1.0; primer3.ut.ee) and assessed for specificity using the Primer-BLAST tool of NCBI (www.ncbi.nlm.nih.gov/tools/primer-blast/ (accessed on 7 February 2019)).

### 2.5. Amplification-Refractory Mutation System Assay (ARMS-qPCR)

Primers for a non-discriminating (consensus) and an SNP discriminating amplification-refractory mutation system (ARMS) qPCR assay were designed to further investigate *STAT5B c.1924A>C* (p.N642H), *STAT3 c.1919A>T* (p.Y640F) and *c.1981G>T* (p.D661Y) hotspot mutations. A mismatch at the penultimate position of the SNP was inserted into the ARMS primers to enhance allelic discrimination (Table S2) [34]. Oligonucleotides were designed using NetPrimer (www.premierbiosoft.com/netprimer/ (accessed on 7 May 2020) and assessed for specificity using NCBI Primer-BLAST.

The 20 µL reaction mix of qPCR contained 1 U HOT FIREPol^®^ DNA Polymerase, 1× PCR buffer B2, 3.5 or 4 mM MgCl_2_, (all Solis Biodyne, 01-02-00500, Tartu, Estonia), 0.2 mM dNTP Mix (Thermo Scientific, R0181), 250 nM for consensus primers or 200 nM ARMS primer, 150 or 200 nM double quenched FAM-labelled hydrolysis probe (all Integrated DNA Technologies, BVBA, Leuven, Belgium) and 5 µL (5 ng/µL) of genomic DNA in TE buffer (10 mM Tris-HCl, 0.1 mM EDTA).

The hot start reaction was initialised by incubation for 15 min at 95 °C, followed by a 3-step cycling protocol: denaturation for 15 s at 95 °C, annealing for 25 s at 55–59 °C and elongation for 25 s at 60 °C over 45 cycles. Amplification efficiency and limits of quantification and allelic discrimination of the assays were determined by a standard curve using synthetic dsDNA templates (377–389 bp; Figures S6 and S7 and Supplementary Data), carrying the alternative base for the SNP (Integrated DNA Technologies, BVBA) [35]. DNA isolated from FFPE sections was used as a spike to screen for a potential influence of the test material on the reaction. Serial dilutions of a standard were analysed in triplicate. Reactions for FFPE material and the no-template control were run in duplicate.

Specificity of the primer amplification was controlled by gel electrophoresis on a 2% agarose gel run in a 40 mM Tris, 20 mM acetic acid and 1 mM EDTA (TAE) buffer adjusted to pH 8.0. 4 µL Atlas ClearSight DNA stain (Bioatlas, BH40501, Tartu, Estonia) were used per 100 mL gel and the size was compared to GeneRuler 100 bp plus DNA ladder (Thermo Scientific, SM0321).

After adjusting to an amplification efficiency of 100%, we compensated for the slightly delayed signal of the ARMS assay deducted from its higher *y*-intercept. Subsequently, we calculated the frequency of SNP DNA using the ∆∆Ct method [36].

### 2.6. Statistics and Bioinformatics

Statistical analysis was done in GraphPad Prism^®^ 5 (Graphpad Software, Inc., San Diego, CA, USA). The normality of the data was analysed via the D’Agostino–Pearson test. The non-parametric Kruskal–Wallis test was used to compare the STAT3 and STAT5B stainings between the groups and the control, and a post-hoc Dunn’s multiple comparison test to examine the differences between the individual groups to the control. All values are given as mean ± standard deviation (SD). If not stated otherwise in the figure legend, the following sample number per group were used: *n* = 15 (EATL I), *n* = 17 (EATL II), *n* = 8 (PTCL-NOS), *n* = 2 (other T cell-associated diagnosis). The following *p* values were accepted as statistically significant: * *p* < 0.05; ** *p* < 0.01; and *** *p* < 0.001. Clustal W and NCBI Blast were used for sequence alignments. Potential splicing sites were analysed with Splicing Finder (INSERM) [37]. The data that support the findings of this study are available from the corresponding author upon reasonable request.

## 3. Results

### 3.1. STAT3 and STAT5B Are Activated in Feline Alimentary Lymphoma

**Figure 1:**
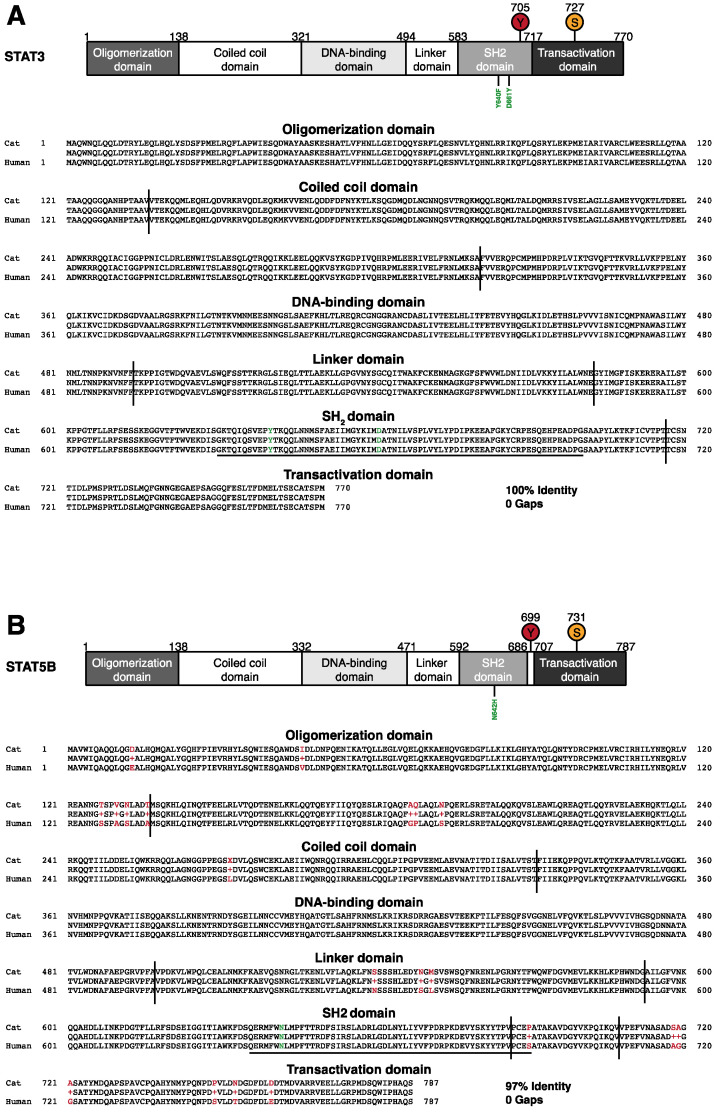
High conservation between feline and human STAT3 and STAT5B. (**A**) Schematic representation of protein domains in STAT3. Top panel: The position of amino acids at the beginning and the end of the respective domains are indicated above the scheme, including tyrosine and serine phosphorylation sites. Two important sites for mutation, Y640 and D661 (marked in green), are shown underneath. Lower panel: Alignment of cat and human STAT3, with protein domains indicated. The amino acid sequence corresponding to the exon sequenced in later analyses (Figure 4) is underlined, Y640 and D661 are marked in green. (**B**) Top panel: Schematic representation of STAT5B as in (**A**). Lower panel: Alignment of cat and human STAT5B, with protein domains indicated. Human and cat STAT5B show 97% identity and no gaps, mismatched amino acids are given in red, N642 is marked in green. The amino acid sequence corresponding to the exon sequenced in later analyses (Figure 4) is underlined.

To analyse the potential involvement of STAT3 and STAT5B in feline EATL and PTCL-NOS, we first examined the homology between the human and feline proteins. Sequence alignment of feline and human STAT3 showed a conserved domain structure (Figure 1A) and the presence of the important tyrosine and serine phosphorylation sites, Y705 and S727. This conservation also applies to amino acids which are frequently mutated in humans (Y640 and D661) and result in constitutive activation. Specifically, a detailed sequence comparison revealed 100% identity of STAT3 between humans and cats (Figure 1A). Likewise, the overall domain organisation is conserved for STAT5B, including the important phosphorylation sites Y699 and S731 (Figure 1B), and the surrounding amino acids. The overall identity at the amino acid level is 97% with no gaps, and the asparagine 642 (N642), an important mutation site in the context of intestinal T cell lymphoma, is also conserved.

**Figure 2:**
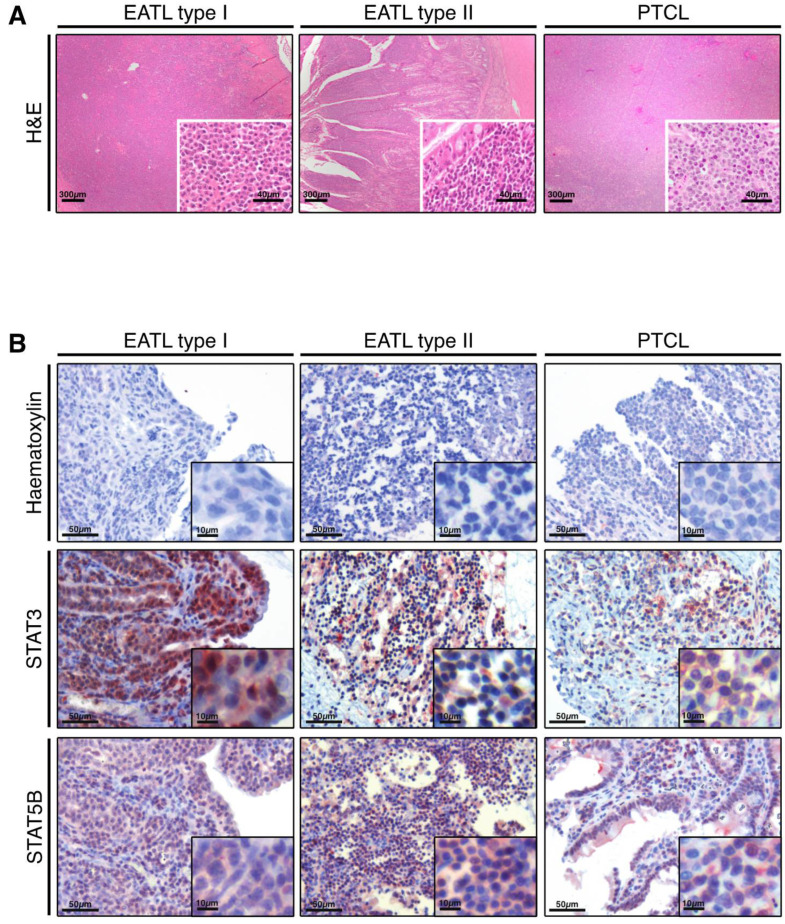
Expression and predominant nuclear localisation of STAT3 and STAT5B in feline alimentary lymphoma cells. (**A**) Haematoxylin and eosin (H&E) stained sections of feline intestine (left and middle) and mesenteric lymph node (right) with diffuse infiltration of lymphoid cells. Lymphoma are classified as EATL type I (left): transmural infiltration of intestine with large lymphoid cells, bar = 300 µm, Insert: bar = 40 µm), EATL type II (middle): mucosal infiltration of intestine with small lymphoid cells, bar = 300 µm; Insert: bar = 40 µm) and PTCL (right): diffuse infiltration of lymph node with intermediate to large-sized lymphoid cells, bar = 300 µm, Insert: bar = 40 µm). (**B**) Tumour cells identified as in (**A**) are used for protein detection. First row: Haematoxylin staining to define nuclei of tumour cells. Second Row: Detection of STAT3 by immunohistochemistry. Third row: Detection of STAT5B by immunohistochemistry. Staining of nuclei indicates activated STAT3 or STAT5B signaling. Representative images of 42 samples are shown.

The complete or high sequence identity between feline and human STAT3 and STAT5B suggests that both proteins fulfil comparable biologic functions, are activated by the same kinases and are deactivated by the same tyrosine phosphatases. Thus, we reasoned that they are potentially also subject to the same activating mutations. Since the activation of both proteins is implicated in the development of human peripheral T cell neoplasia (Figure S1), we wanted to examine if the same is true for felines where these diseases occur at a higher frequency. To this end, we tested monoclonal antibodies recognising human and murine STAT3 and STAT5B protein on paraffin-embedded sections of feline and murine liver tissue. We observed consistent staining patterns between both species (data not shown), enabling the analysis of feline tumour tissue from EATL type I, EATL type II and PCTL-NOS patients (Figure 2A). STAT3 is expressed strongly in all cases of the three tumour types (Figure 2B). Strikingly we observed a very strong nuclear staining, suggesting a high level of activation [38]. STAT5B also shows high expression levels in all cases of the three tumour types, and a similar trend in the ratio of nuclear versus cytoplasmic localisation as seen for STAT3, again suggesting strong activation of STAT5B (Figure 2B) [39]. We used sub-cellular localisation of STAT proteins as a reliable activation marker since oxidation processes of long-term stored paraffin-embedded specimens can result in artifacts upon pY-STAT staining [38].

**Figure 3:**
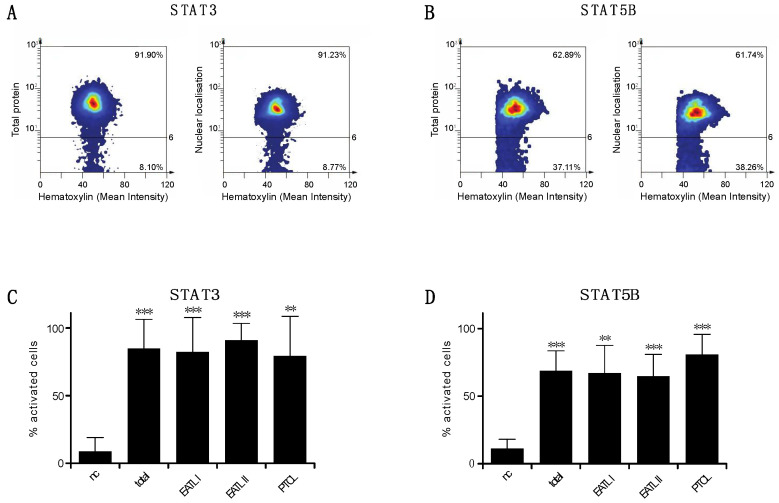
STAT3 and STAT5B are activated in feline alimentary lymphoma cells. (**A**,**B**) Cytometric analysis of tumour tissue stained for STAT3 and STAT5B by IHC as in Figure 2. Nuclei and cytoplasm of individual cells were determined by image analysis software as in Figure S2. Staining intensity of the indicated immunohistochemical marker was determined for each individual cell and for both cellular compartments, representative samples are shown. (**A**) Analysis of total cellular STAT3 in feline tumour cells. Right panel: Nuclear localisation of STAT3 in tumour cells. (**B**) Analysis of total protein content and nuclear localisation of STAT5B as in A. (**C**) Percentage of tumour cells showing activation of STAT3 as determined by the ratio of nuclear versus cytoplasmic staining. (**D**) Percentage of tumour cells showing activation of STAT5B as determined by the ratio of nuclear versus cytoplasmic staining. For both markers, activation was determined in total and for samples classified as EATL I, EATL II and PTCL-NOS. nc = negative control (normal feline liver tissue, Figure S2I), *n* = 15 (EATL type I), 17 (EATL type II) and 8 (PTCL-NOS), ** *p* < 0.001, *** *p* < 0.0001; error bars = SD.

The nuclear localisation of STAT3 and STAT5B observed in EATL type I, type II and PTCL-NOS tumour cells prompted us to analyse whether this is a recurrent phenomenon. We collected feline samples from a larger cohort, representing 15 cases of EATL type I, 17 cases of EATL type II and 8 cases of PTCL-NOS and subjected them for immunohistochemistry as before. To analyse the activation status of STAT3 and STAT5B in an unbiased manner, an automated image analysis system (HistoQUEST, TissueGnostics GmbH) was used to determine and calculate the ratio of the respective protein in the cytoplasm versus the nucleus (Figure S2). Tissue cytometry revealed high expression of STAT3 in virtually all of the alimentary tumour cells, as exemplified in a representative sample (Figure 3A, left panel). A large fraction of the detected protein is present in the nucleus, a hallmark for the activation of STAT proteins, as indicated in Figure 3A (right panel). Comparable to STAT3, STAT5B is also expressed by a large fraction of the tumour cells, and a high proportion of the protein is located in the nucleus (Figure 3B). A statistical analysis of all 42 tumour samples, including EATL I, EATL II and PTCL-NOS, revealed the activation of STAT3 in 82.35% of all tumour cells examined, as determined by the ratio between cytoplasmic and nuclear localisation. Specifically, STAT3 is activated in 78.88% of tumour cells from all EATL I samples examined, in 87.32% of EATL II samples, and in 78.93% of PCTL cases (Figure 3C). Similarly, STAT5B is activated in 61.46% of total tumour cells examined, and in 61.46% of EATL I, 59.3% of EATL II and 75.08% of PTCL-NOS cases (Figure 3D). Overall, we conclude that STAT3 and STAT5B are expressed in practically all tumour cells of the feline EATL I, EATL II and PTCL-NOS samples examined and show a high level of activation in these cells.

### 3.2. Conservation of the Genomic Locus and the Intron-Exon Structure of Feline STAT3 and STAT5B

**Figure 4:**
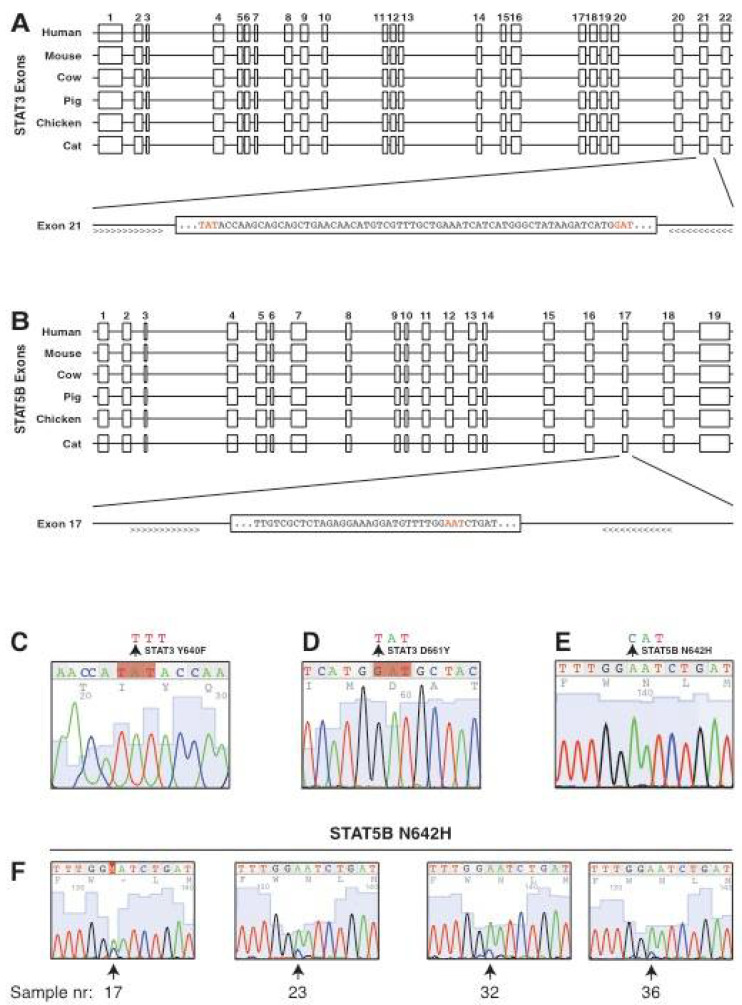
Conservation of the intron–exon structure of *STAT3* and *STAT5B* and screening for candidate activating mutations. (**A**) Schematic representation of the sequence alignment of *STAT3* from the indicated species. Exons are drawn as boxes with their numbers given above, introns as lines. The lower panel is a close-up of *exon 21* from the genomic sequence of feline *STAT3*. The sequence shows that two codons (red) representing mutational hotspots in human and murine *STAT3* are conserved in feline *STAT3*. (**B**) Schematic representation of the sequence alignment of *STAT5B* from the indicated species as in A. Exons are drawn as boxes with their numbers given above, introns as lines. The lower panel is a close-up of *exon 17* from the genomic sequence of feline *STAT5B*. The sequence in the box represents part of *exon 17* surrounding the codon corresponding to N642, which is also present in feline *STAT5B*. (**C**–**E**) Examples of Sanger sequencing of nucleotides encoding for the amino acids Y640 (**C**) and D661 (**D**) of *STAT3* and N642 (**E**) of *STAT5B*. Arrows indicate the position of the nucleotide that is changed in known activating mutations (Y640F: TAT → TTT, D661Y: GAT → TAT, N642H: AAT → CAT). Primers as indicated in A were used to amplify *exon 21* of *STAT3* (**C**,**D**) and *exon 17* of *STAT5B* (**E**). (**F**) Results from Sanger sequencing of lymphoma samples for the N642H mutation of *STAT5B*. Indicated samples show minor peaks for the N642H mutation of *STAT5B*, corresponding to an A to C conversion. Position and sample number are indicated by an arrow below each panel.

The activation of STAT3 and STAT5B in feline alimentary T cell lymphomas is comparable to the human disease, therefore, we continued to analyse the genomic status of the respective *STAT* genes. *STAT3* and *STAT5A* are in close proximity and arranged in a head-to-tail configuration, whereas *STAT5B* is more distant and shows the same orientation as *STAT3*. This arrangement is conserved among all species of veterinary interest examined, except for chicken. However, we assume that the comparably low sequence coverage and associated difficulties in annotation are likely responsible for this exception (Figure S3). All exon-intron boundaries of feline *STAT3* are conserved between human and murine *STAT3*, which also applies to cattle, pig and chicken (Figure 4A). *Exon 21* of human and murine *STAT3* harbours two sites for mutational activation, Y640F and D661Y (Figure 4A), both of which are identified as driver mutations in EATL and PTCL-NOS [27,28]. To determine the sequence of feline *exon 21*, we designed feline-specific primers targeting the *exon 21*-surrounding introns. Furthermore, in the case of *STAT5B*, the number of exons and all exon-intron boundaries are conserved between the indicated species. The main activating mutation for *STAT5B*, N642H, is located in *exon 17* corresponding to the SH2 domain of the protein. To determine the potential presence of the same activating mutations as in humans, we designed primers flanking *exon 17* of the cat (Figure 4B). A detailed comparison between human and feline *STAT3* or *STAT5B* revealed the highest sequence identity of 94% and 93% in the coding sequence, with a decline to 82% and 83% in the relatively short 5′-untranslated region (UTR) and the weakest sequence conservation of 73% and 72% in the 3′-UTR (Figures S4 and S5).

### 3.3. Screening for Candidate Mutations of STAT3 and STAT5B in Feline Alimentary Lymphoma

**Figure 5:**
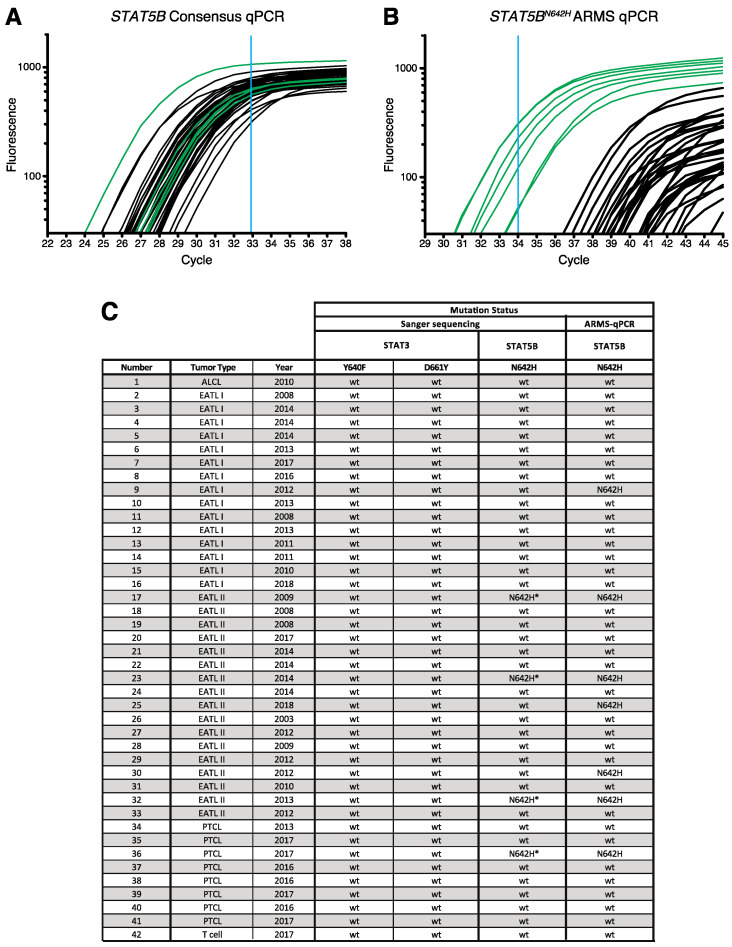
Confirmation of known activating mutations of *STAT5B* in feline alimentary tumour cells by ARMS-qPCR. (**A**,**B**) Amplification plot obtained using the *wild-type* (consensus) primers (**A**) and the *STAT5B^N642^* mutant-specific (ARMS) primers (**B**) in qPCR with genomic DNA of the alimentary lymphoma samples indicated in (**C**). Samples showing a positive signal in the ARMS-qPCR are indicated in green in panels (**A**,**B**). Fluorescence is presented as fluorescence intensity over background, and (**A**,**B**) have been corrected for the standard curves in Figure S6. Blue lines indicate the lower limit of the linear dynamic range of the assays. *n* = 3 each. (**C**) Table showing the samples used in the analysis, their pathological classification and the year of isolation together with the results from Sanger sequencing for Y640 and D661 of *STAT3* and for N642 of *STAT5*. N642H * indicates a minor peak in the sequencing reaction as shown in Figure 4F, corresponding to N642H. ALCL = anaplastic large cell lymphoma.

The activation of STAT3 and STAT5B in feline alimentary lymphoma and the conservation of these genes at the genomic level prompted us to analyse the potential presence of activating mutations already identified in the corresponding human diseases. Genomic DNA was isolated from the same EATL type I, EATL type II and PTCL-NOS patient samples that were used to determine the activation of STAT3 and STAT5B (Figure 2 and Figure 3). Primers depicted in Figure 4A,B were used to amplify *exon 21* of *STAT3* and *exon 17* of *STAT5B*. Sanger sequencing revealed the *wild-type* status in representative examples for the codons encoding Y640 and D661 for *STAT3*, and N642 for *STAT5B* (Figure 4C–E). *STAT3* did not show any detectable changes in bases corresponding to known activating mutations, nor in any other bases of *exon 21*. In the case of *STAT5B*, four samples displayed minor bands for the N642H mutation together with the *wild-type* sequence (Figure 4F and Figure 5C). Re-amplification and sequencing resulted in varying signal strength for the *STAT5B^N642H^* mutation, indicating a potential inherent bias or randomness in the Sanger sequencing reaction. Besides these *STAT5B^N642H^* mutations, no other changes were detected in *exon 17* of *STAT5B* in all samples tested.

### 3.4. Confirmation of STAT5B N642H Mutation in Feline Alimentary Lymphoma Cells

To determine if peaks detected in the Sanger sequencing reaction represented real signals for *STAT5B^N642H^*, we performed an independent measurement of the target mutation at higher resolution and better quantitative accuracy using the amplification refractory mutation system-quantitative polymerase chain reaction (ARMS-qPCR) [34]. Calibration curves using consensus and ARMS primers exhibited high linearity over a wide range of target concentrations (Figure S6), and were used for normalisation (Figure 5A,B). According to the regression analysis (Figure S6), the cut-off point was set at a *Cq* value of 32.8. Analysis of the 42 samples of feline alimentary lymphoma using the consensus primers in qPCR revealed the range of detection of the *wild-type* sequence, indicating the presence of the *wild-type* sequence in all samples. The ARMS-qPCR identified seven samples as positive for the *STAT5B^N642H^* mutation, according to the cut-off *Cq* value of 34.1 (Figure 5B,C). We also analysed the 42 tumour samples for the presence of *STAT3^Y640F^* and *STAT3^D661Y^*, using a single consensus primer set covering both sites and individual allele-specific primers. Calibration curves for the consensus and mutation-specific primers (Figure S7A) revealed linearity over four log_10_ orders of magnitude of the target concentration. While the consensus primers recognised their *wild-type* target within the linear range in all 42 cases (Figure S7B), none of the samples reached the limit of quantification when targeting the mutations (Figure S7C,D). In summary, the ARMS-qPCR confirmed the presence of the *STAT5B^N642H^* mutation detected by Sanger sequencing and revealed further positive cases (Figure 5C).

### 3.5. No Enrichment for Infection with Feline Leukaemia Virus (FeLV) in Patient Samples and Identification of a Novel Polymorphism of STAT5B

**Figure 6:**
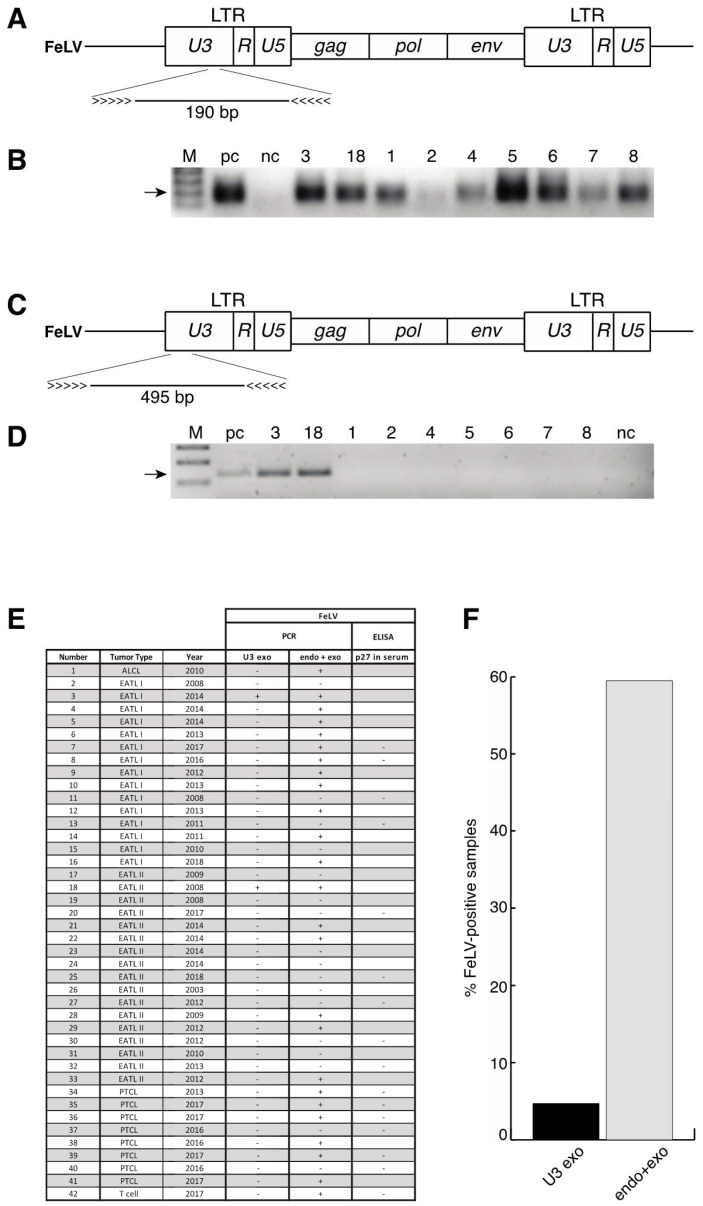
No accumulation of active FeLV among tumour samples. (**A**) Scheme of FeLV structure and location of the primers in the *U3* region used for detection of endogenous and exogenous virus sequences. (**B**) Representative images of amplifications of FeLV sequences using primers shown in (**A**). pc = positive control (tissue from a feline patient that was tested positive for FeLV p27 by ELISA) nc = negative control (tissue from a feline patient that was tested negative for FeLV p27 by ELISA) (**C**) Scheme of FeLV structure and location of the primers in the *U3* region used for detection of exogenous virus sequences. (**D**) Representative images of amplifications of FeLV sequences using primers shown in (**C**). pc and nc as in (**B**). (**E**) Table summarising the results from the PCR analysis and ELISA. *U3 exo* corresponds to the detection of exogenous FeLV sequences as in (**C**,**D**), endo + exo to the detection of endogenous and exogenous sequences as in (**A**,**B**). The status of p27 was detected in the serum of patients by ELISA as shown in the last row. (**F**) Percentage of samples testing positive for exogenous (*U3 exo*), exogenous and endogenous (endo + exo) FeLV by PCR or for p27 by ELISA among the 42 samples of the cohort. The original images of (**B**,**D**) can be found in Figure S9.

FeLV is a gamma retrovirus that causes a complex disease spectrum encompassing lymphoid lymphoma. The infection rate with FeLV has dropped since the development of a vaccine against FeLV, however, there could be a selective enrichment for FeLV-infection among the samples tested [40]. We determined the infection rate of the host’s DNA with the provirus by PCR in order to examine all samples. Over time, strains of FeLV have entered the feline germline and have become incompetent of viral replication. Therefore, we designed primers recognising part of the *U3* region of the long terminal repeats that do not distinguish between endogenous, replication-deficient and exogenous, replication-competent viruses (Figure 6A). Using this strategy, we detected proviral sequences in the genome of the feline samples (Figure 6B). In addition, we used published primers designed against part of the *U3* region of the FeLV long terminal repeat that is specific for replication-competent, exogenous virus (Figure 6C). A representative analysis shows amplifications also for exogenous FeLV, albeit at a much lower rate compared to the first primer set (Figure 6D). Analysis of all samples revealed a low infection rate with exogenous, actively replicating viruses, with only two samples testing positive among a total of 42, representing 4.74% (Figure 6E,F). Conversely, the rate of samples tested positive for endogenous and exogenous FeLV was at 59.52% of all samples (Figure 6E,F). ELISA testing for the p27 protein was performed in 16 of the 42 samples, and all of them were negative for FeLV-derived p27, corresponding to the data using exogenous-specific primers (Figure 6E).

The analysis of *exon 21* of *STAT3* and *exon 17* of *STAT5B* also included intronic sequences, upstream and downstream of both exons. Surprisingly, the intronic sequence upstream of *exon 17* of *STAT5B* displayed a mismatch to the published genome sequence (Felis catus assembly 9.0; Figure S8). The canonical sequence occurred in 7% of the 42 samples, the newly discovered polymorphism in 74% and the heterozygous situation in 19% (Figure S8B). Screening of the available databases for single nucleotide polymorphisms (SNP) of the cat genome revealed no match. Since the samples in this study are almost exclusively derived from European Shorthair breed, a strain that is not included in the cat genome project, we report on a novel SNP. The polymorphism is located at position −25 relative to the beginning of *exon 17* (Figure S8C). This is in proximity to the first potential branching point for the upstream splice donor at position −22 (Figure S8C). Interestingly, the canonical sequence represents a predicted binding site for hnRNP1, a protein that is involved in splice site selection, and this motif is lost in the new polymorphism (Figure S8D). STAT5B undergoes differential splicing and proteolytic processing, but most studies have concentrated on the full-length protein or its complete loss. In further studies, it will be interesting to determine potential new splice variants and their function.

## 4. Discussion

Rare diseases mostly represent a serious, life-threatening and debilitating group of disorders. Human intestinal lymphomas are rare diseases with an incidence of one per 1,000,000 inhabitants per year [41,42]. This paucity poses a major challenge in unravelling their underlying mechanism and developing standard therapeutic strategies [43,44]. The unique spectrum of naturally-occurring cancers in the cat offers significant opportunities for comparative and translational advances that may have mutual benefit for human and veterinary medicine. The study of feline cancers additionally may generate new insight into underexplored aspects of tumour biology that are less accessible in other species. Alimentary lymphoma, which comprises more than 50% of all feline lymphomas, remains vastly understudied at the molecular level [45,46].

Here, we discover a striking conservation of the feline and human STAT3 and STAT5B protein sequence. STAT3 is completely identical in these two species. STAT5B shares 97% sequence identity with conservation of all known sites for phosphorylation, the surrounding amino acids as well as the SH2 domain, the major site for activating mutations in STAT proteins [47,48]. This high sequence conservation, which also extends to JAK kinases (data not shown), likely indicates functional conservation. Indeed, human interleukin-2 is able to sustain the proliferation and survival of feline T cells [49,50]. Both proteins, STAT3 and STAT5B, are activated in virtually all of the alimentary lymphoma samples examined. Therefore, independent of the potential presence of a mutation of these genes, this offers the possibility of pathway interference using established inhibitors of JAK kinases or newly developed inhibitors of STAT3 or STAT5B [51]. Furthermore, it is another parallel to the corresponding tumours in humans, where both proteins can be hyper-activated.

The genomic structure of *STAT3* and *STAT5B* is conserved between felines and several other vertebrate species, with the same intron-exon structure and potential sites of mutations being in the corresponding exon to their human counterparts. This conservation allowed targeted sequencing of *STAT3 exon 21,* encompassing the mutation residues Y640 and D661, and *exon 17* of *STAT5B*, encoding the commonly mutated N642H. Mutations at all of these sites result in constitutively active proteins and have been implicated in human gastrointestinal lymphoma [25,27,28]. Together with allele-specific quantification performed by ARMS-qPCR, we were able to identify seven cases of *STAT5B^N642H^* out of a total of 42 cases (16.67%). The 42 samples comprise of 17 cases of EATL type II, to which five of the *STAT5B^N642H^* mutations belong (29.41%), which is comparable to the range that has been reported for human MEITL [26,28]. One *STAT5B^N642H^* mutation falls into the feline EATL type I category and one into PTCL-NOS. This could reflect differences in the classification of enteropathy-associated lymphomas in the cat, as fewer reagents like antibodies are available [3,9]. Moreover, human patients with EATL or PTCL-NOS have also been found positive for *STAT5B^N642H^*, although this is much rarer than in MEITL patients [28,29]. In addition, the long-term storage of the samples might affect the detection efficiency of the mutant allele to some degree. Interestingly, activation of JAK/STAT signaling has also been described in human ITCL, including activation of STAT5, although the *STAT5B^N642H^* mutation has not been detected so far [52,53].

The identification of *STAT5B^N642H^* in feline and human intestinal lymphoma by two independent methods has several implications. The most important consequence is that human and feline intestinal lymphomas have at least in part a shared genomic cause, thus establishing feline intestinal lymphoma for comparative human studies. Interestingly, the human lymphoma subtypes that have been linked to feline EATL type II, MEITL and ITCL, show activation of JAK/STAT signaling. It will be interesting for further studies to determine if *STAT5B^N642H^* also occurs in human ITCL and if feline EATL type II can be further subdivided. *STAT5B^N642H^* is the most frequent mutation in MEITL described so far, however, it is not the only one [26,27]. Recurrent mutations in human MEITL include JAK kinases, potentially triggering the activation of STAT3 and/or STAT5 oncoproteins, the constituents of the MAP kinase pathway (BRAF, KRAS), chromatin modifier genes (SETD2, CREBBP), and a G-protein-coupled receptor gene (GNAI2). It will be interesting for further studies to determine if other recurrent mutations occurring in human MEITL are found in felines as well.

Inhibitors of JAK/STAT signaling offer interesting therapeutic perspectives, as the activation of STAT3 and STAT5B was observed in virtually all cases of feline alimentary lymphoma. However, these inhibitors should be specific for STAT3 and STAT5B, as other members of the STAT family are involved in important immune reactions [21,47,51]. New inhibitors targeting the SH2 domain of STAT3 and STAT5 are currently under development and should provide the possibility of interfering with the respective proteins in a specific manner [54,55]. Since *STAT5B^N642H^* has been identified as a driver of various T cell neoplasia, particularly of γδ T cell lymphoma, such a targeting approach is likely to be beneficial in these diseases [56]. The availability of a host with a high prevalence of intestinal T cell lymphomas will particularly benefit such translational studies. The establishment of cell lines from feline patients with the *STAT5B^N642H^* mutation is the next step in exploring the potential of feline lymphoma cells for comparative aspects of the human disease. This will offer the possibility for further elucidating the mechanistic basis of these diseases and for screening and testing of this novel class of STAT3 and STAT5B inhibitors in a physiologically relevant setting.

Two different causes for feline lymphoma are currently recognised, retrovirus-associated and non-retroviral in origin. Feline leukaemia virus is the most lymphomagenic of the feline retroviruses, with infected cats having a 60-fold increased risk of developing cancer [57]. We excluded the possibility of FeLV being overrepresented in our samples by two different PCR strategies, confirming a high prevalence of endogenous, most likely replication-incompetent viruses in the genome of European short-hair cats, whereas infections with active FeLV were detected at a reported low frequency [58,59]. Thus, our study does not reflect the potential influence of a feline-specific retrovirus on lymphomagenesis and a potentially altered mutational spectrum.

In summary, we demonstrate the activation of STAT3 and STAT5B in feline alimentary tumour cells, and identify the activating mutation *STAT5B^N642H^* in feline EATL type II, as frequently observed in human MEITL patients. This offers the potential of using this relatively frequent feline tumour type for comparative studies of the human disease and as a new tool for the screening and development of novel therapeutic approaches for this rare and incurable human disease.

## 5. Conclusions

Enteropathy-associated T cell lymphomas (EATL) are rare and fatal human diseases, understudied due to their paucity. However, they occur more frequently in felines, offering the potential to establish a naturally occurring model for a rare human disease. Here, we identify for the first time the activating driver mutation *STAT5B^N642H^* in feline alimentary lymphoma. We observe strong activation of STAT5B and STAT3 at the protein level and report the absence of common activating mutations of STAT3, which is again comparable to the human disease. Overall, this data describes a comparative oncogenic driver mutation occurring in a companion animal without the need for genetic engineering or chemical induction. Thereby, it lays the foundation to establish a novel biologically relevant model.

## Data Availability

The data presented in this study are available on request from the corresponding author.

## Supplementary Materials

The following are available online at https://www.mdpi.com/article/10.3390/cancers13205238/s1, Figure S1: Depiction of somatic mutations detected in human STAT5A, STAT5B and STAT3 in patients with hematopoietic cancers. Figure S2: Determination of protein localisation by image analysis software and antibody control. Figure S3: Comparison of genes in the Stat3/5 locus in various species. Figure S4: Comparison of conserved regions between human and feline *STAT3*, Figure S5: Comparison of conserved regions between human and feline *STAT5B*, Figure S6: Standard calibration curves for primer pairs detecting the *wild-type* (consensus) sequence of *STAT5B^N642^* and the mutant (ARMS) sequence of *STAT5B^N642H^* Figure S7: Analysis of known activating mutations of *STAT3* in feline alimentary tumour cells by ARMS-qPCR. Figure S8: Identification of a novel polymorphism in a potential splice site of feline *STAT5B.* Figure S9: Original Agarose Gels. Table S1: Details of feline lymphoma sample cohort, Table S2: Oligonucleotides for PCR, qPCR and ARMS-qPCR assays, Supplementary Data: Template dsDNA fragments for ARMS qPCR.
