# Supplementary material for: A Recurrent STAT5BN642H Driver Mutation in Feline Alimentary T Cell Lymphoma
Source: Cancers (Basel). 2021 Oct 19;13(20):5238. doi: 10.3390/cancers13205238 (PMC8534107; doi:10.3390/cancers13205238)
Supplement: Supplementary file 1 [file cancers-13-05238-s001.zip › cancers-1404868-supplementary after proof new.pdf]

cancers

Supplementary Material: A Recurrent *STAT5B*^{N642H} Driver Mutation in Feline Alimentary T Cell Lymphoma

Matthias Kieslinger, Alexander Swoboda, Nina Kramer, Patricia Freund, Barbara Pratscher, Heidi A. Neubauer, Ralf Steinborn, Birgitt Wolfesberger, Andrea Fuchs-Baumgartinger, Richard Moriggl and Iwan A. Burgener

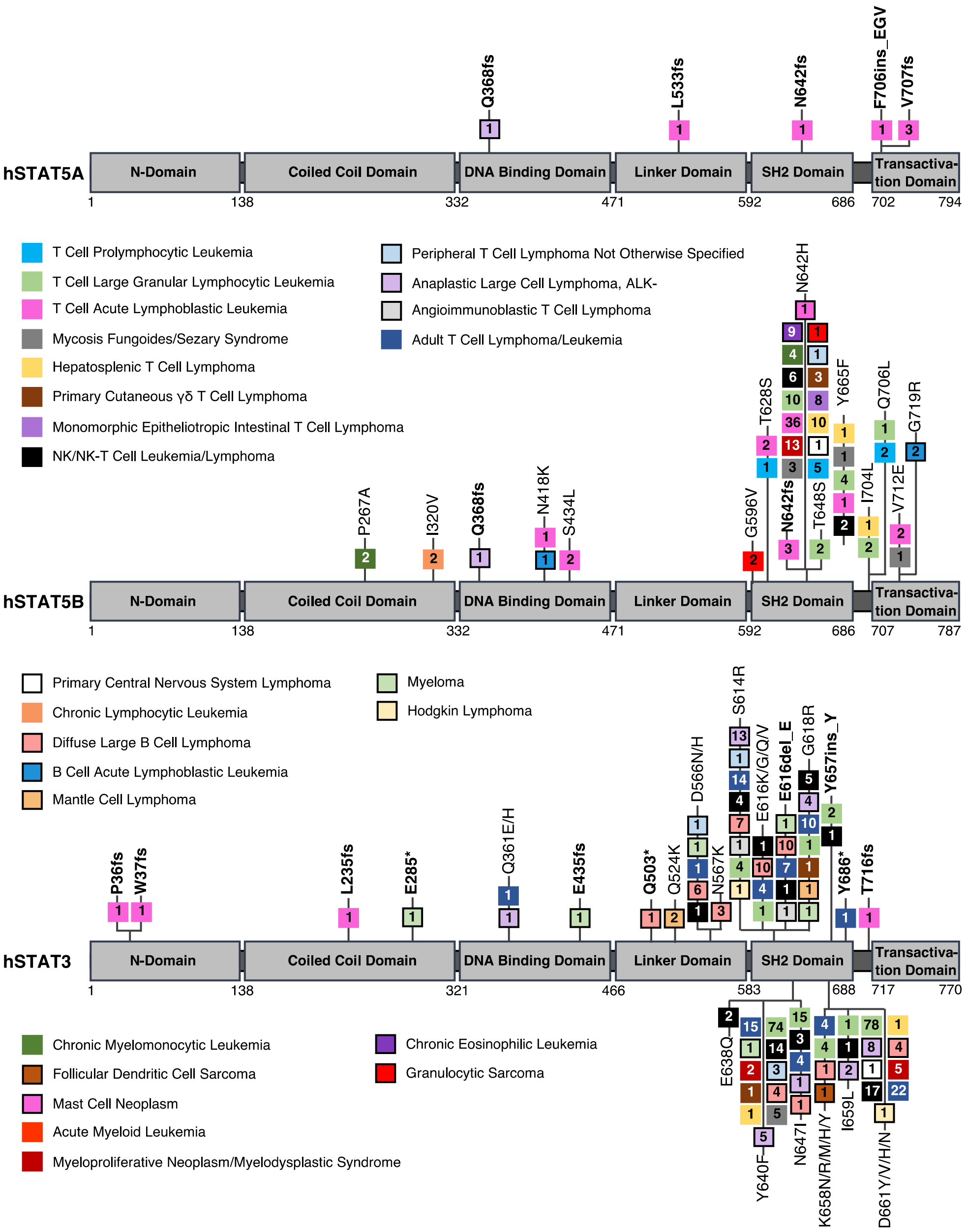

Figure S1. Depiction of somatic mutations detected in human STAT5A, STAT5B and STAT3 in patients with hematopoietic cancers. Individual missense mutations found in at least two patients, as well as all reported nonsense and frameshift mutations (bold), are depicted. Numbers in each box represent the number of cases reported for each mutation. Data were mined from the Catalogue of Somatic Mutations in Cancer (COSMIC) database. SH2, Src homology 2.

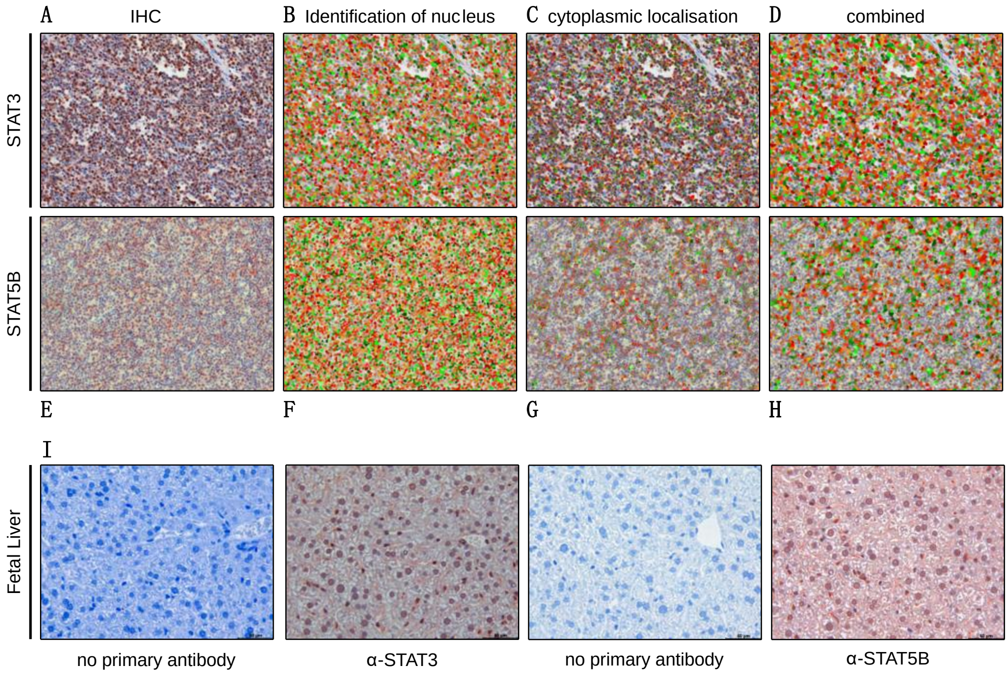

Figure S2. Determination of protein localisation by image analysis software and antibody control. Tissue sections of EATL type II were stained by immunohistochemistry for STAT3 (A-D) or STAT5B (E-H) and counterstained by haematoxylin to visualise nuclei (A, E). Nuclei were identified by computer-based image analysis (HistoQuest 6.0; TissueGnostics) based on haematoxylin (B, F). Different colours were used to distinguish between different nuclei. Cytoplasm was determined in a second step, and overlayed with colour intensity information from immunohistochemistry (C, G). Colours in the middle right panel do not indicate staining intensity, but represent the cytoplasm of individual cells positive for STAT3 or STAT5B. Nuclei were overlaid with data from immunohistochemistry as for cytoplasm (D, H) to quantify the respective protein. (I) Tissue sections of fetal liver were stained with the indicated antibodies or for control without primary antibody. The localisation was used for normalisation of Fig.3C and D.

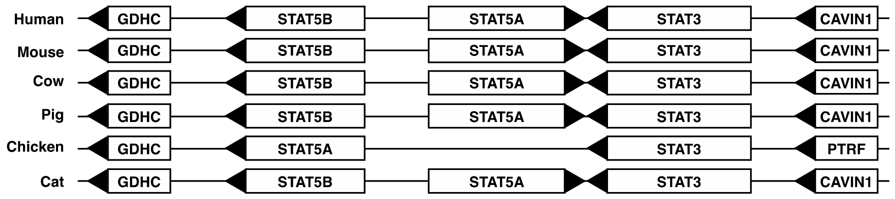

Figure S3. Comparison of genes in the Stat3/5 locus in various species. Black triangles indicate the direction of transcription, flanking genes are shown. (Co-ordinates: Human: Chr. 17 42,199,177-42,388,373; Mouse: Chr. 11 100,671,557-100,830,366 ; Cow: Chr. 19 42,319,170-42,491,771; Pig: Chr. 12 20,407,233-20,574,288 ; Chicken: Chr. 27 7,694,496-7,727,343; Cat: Chr. E1 42,838,788-42,968,081; all co-ordinates span the STAT genes from 5′ to 3′, co-ordinates are derived from ENSEMBL).

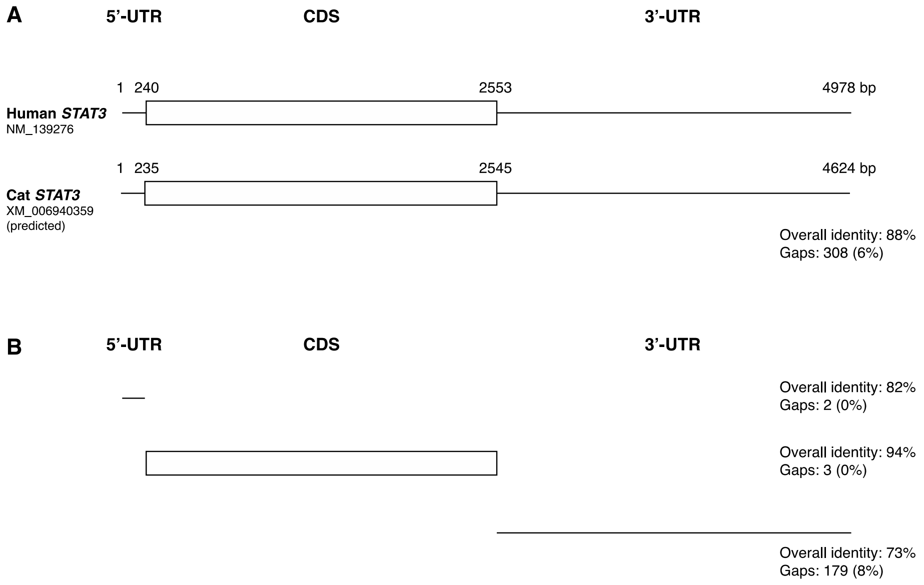

Figure S4. Comparison of conserved regions between human and feline *STAT3*. (A) Schematic representation of human *STAT3* mRNA, with untranslated regions (UTR) depicted as lines and the coding sequence (CDS) as a box. The total length as well as the nucleotide positions of the boundaries are shown above. Overall identity and homology gaps are indicated as well as the NCBI sequence identifiers. (B) Overall identity and homology gaps are shown for 5'-UTR, coding sequence and 3'-UTR.

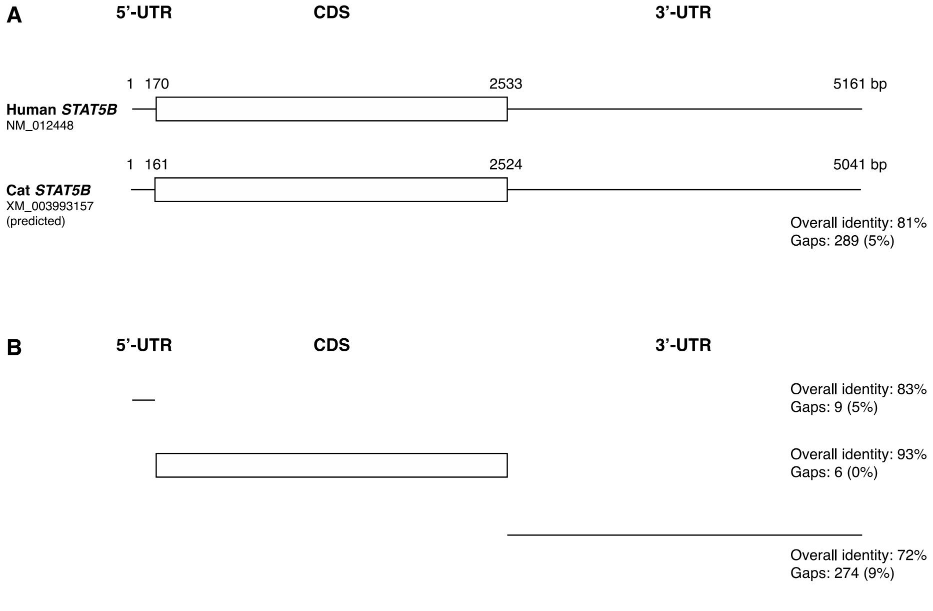

Figure S5. Comparison of conserved regions between human and feline *STAT5B*. (A) Schematic representation of human *STAT5B* mRNA, with UTR regions depicted as lines and the coding sequence as a box. The total length as well as the nucleotide positions of the boundaries are shown above. Overall identity and homology gaps are indicated as well as the NCBI sequence identifiers. (B) Overall identity and homology gaps are shown for 5'-UTR, coding sequence and 3'-UTR.

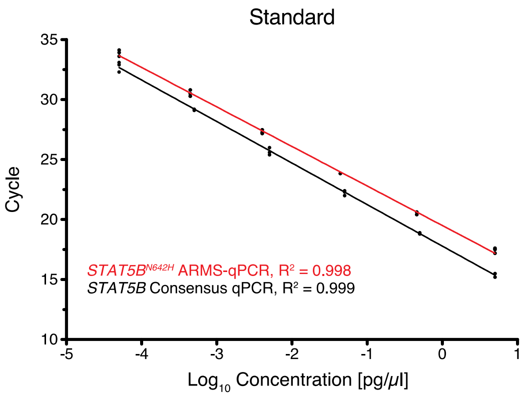

Figure S6. Standard calibration curves for primer pairs detecting the *wild-type* (consensus) sequence of *STAT5B*^{N642} and the mutant (ARMS) sequence of *STAT5B*^{N642H}. Standard calibration curves were generated by plotting the *Cq* values versus the decadic logarithm of diluted standard for *wild-type* and mutant DNA copy number. Amplification efficiency was calculated from the slope of the curve. n = 3.

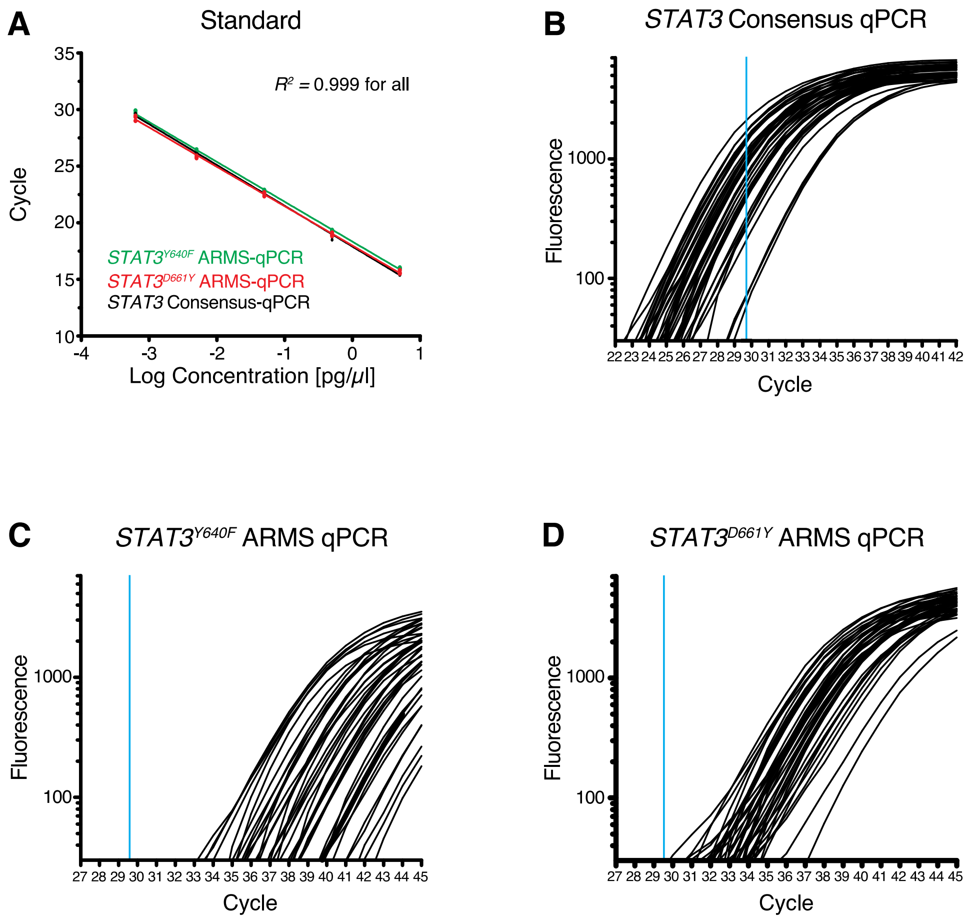

Figure S7. Analysis of known activating mutations of *STAT3* in feline alimentary tumour cells by ARMS-qPCR. (A) Standard calibration curves for primer pairs detecting the *wild-type* (consensus) sequence of *STAT3*^{Y640} and *STAT3*^{D661} on exon 21, the mutant (ARMS) sequence of *STAT3*^{Y640F} and the mutant (ARMS) sequence of *STAT3*^{D661Y}. Standard calibration curves were generated by plotting the *Cq* values versus the decadic logarithm of diluted standard for *wild-type* and mutant DNA copy number. Amplification efficiency was calculated from the slope of the curve. n = 3. (B-D) Amplification plot obtained using the *wild-type* (consensus) primers (B), the *STAT3*^{Y640F} mutant-specific (ARMS) primers (C) and the *STAT3*^{D661Y} mutant-specific (ARMS) primers (D) in qPCR with genomic DNA of the alimentary lymphoma samples indicated in Fig. 5C. Fluorescence is presented as fluorescence intensity over background, and B-D have been corrected for the standard curves in A. n = 3.

A

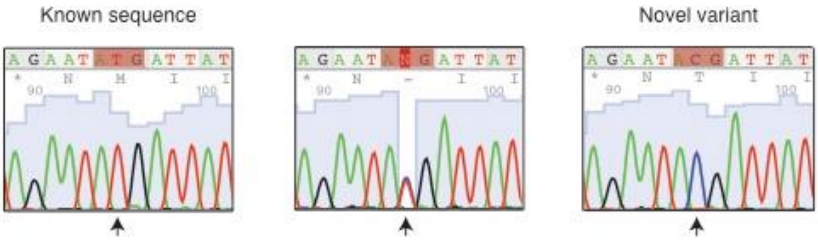

B

	Samples	Percentage
ATG:	3	7%
ATG+ACG	8	19%
ACG:	31	74%
	42	100%

C

```
>chromosome:Felis_catus_9.0:E1:42843971 - 42844894
42844894 AAGATAAGAGTAGCTTGCTAGTTTTTTTGCAACTTAAAGATATTCCCAGCCTTTCACAGC 42844835
42844834 TCTCTGCAGCCTCTGGCTGTCGGCAGGCTTCCTGTCTCACTCAGATGCTTTTGTACAGCA 42844775
42844774 ATCAAGGATTGGAGTCCATAAGAACTATTTCTTCTACTGAGGGACCGAAAAACCATTTGA 42844715
42844714 ACTTGAAACTACCTGAGATCATTGGCTCTCTAGGGAAGTGCCATATGCATTACCTGCTTT 42844655
42844654 TTCCCCCCTGGCTTTGATAAATGACTTGAACTACAACCTGTGGTTAATTTTAAATGGAGA 42844595
42844594 TCTTTATTGGGACAGTTTTAACGGAGAGTCTTTAATTTGTAGCATGTTTGGATTTTTTAA 42844535
42844534 CATTTCCTGTTCAGAAATCATGTTTAGAATACGATTATTCTGTTTGTCGCTCTAGAGGAA 42844475
42844474 AGGATGTTTTGGAATCTGATGCCTTTTACCACCAGAGACTTCTCCATCCGGTCCCTGGCT 42844415
42844414 GACCGCTTAGGGGACCTGAATTACCTTATCTACGTGTTTCCTGATCGGCCAAAAGATGAA 42844355
42844354 GTGTACTCCAAATACTACACACCAGTCCCATGCGAGCCTGCCACTGGTAACAATGTTCGC 42844295
42844294 ATTCTGATCTGGTTTTGTTGCGATTTGAGTTTTCTTTTGGTTTTTGTGTGGGTGCGTGTG 42844235
42844234 TGTGTGTGTGTGTGTTGTGTGTGTGTCTGTCTGTCTTGCATGAAAACCTACACAAT 42844175
42844174 CAGTATTTAGTTATTGCTGTTTGAGTATTATTCAAGGCAGCCGTAAATCCTTGGCTATCT 42844115
42844114 TCTTCAGTCATATAGAAAACATATCTGCTGCTGACCCCAAATATCAAATTGAAGGGAACA 42844055
42844054 AATTTATTATCTTTTTTCGCATTGCTTTTATTTATTCATTTTTTAATGTTTTATTTATTT 42843995
42843994 TGAGAGAGAGAGAGAGAGAGCACA                                     42843971
```

D

Sequence position	Reference sequence	Mutant sequence	Variation
-21	TATGAT	TACGAT	broken
Linked hnRNP protein	Reference motif (value 0-100)	Mutant motif (value 0-100)	Variation
hnRNP1	TATGAT (71.43)	TACGAT (-71.43)	0%

Figure S8. Identification of a novel polymorphism in a potential splice site of feline *STAT5B*. (A) Results of sequencing of *intron 16* of feline *STAT5B*. Primers as depicted in Fig. 4 were used, covering part of the intron preceding *exon 17*. Pictograms show the results from three different individuals harbouring the known sequence as deposited in the feline genome database (left panel), and a novel polymorphism in the heterozygous (middle panel) and homozygous state (right panel). (B) Frequency of the polymorphism among the tested samples. The number and percentage of each state of the polymorphism among the cohort of feline patients is shown. (C) Schematic depiction of the genomic locus surrounding *exon 17* of feline *STAT5B*. *Exon 17* is underlined and bold, the sequence used for analysis is shown in red. Three nucleotides comprising the novel polymorphism are given in blue, known polymorphisms in green, and the triplet encoding for N642 in black. (D) Analysis of the novel polymorphism by functional prediction software (Splicing Finder, INSERM). The position of the polymorphism is shown with respect to the beginning of the following exon (sequence position), the sequence is shown (reference sequence), and the newly identified SNP (mutant sequence). hnRNP1 is identified as a binding protein (linked hnRNP protein), an arbitrary binding value to the cognate sequence (reference motif; 71.43) and its predicted loss in the new polymorphism (mutant motif; -71.43).

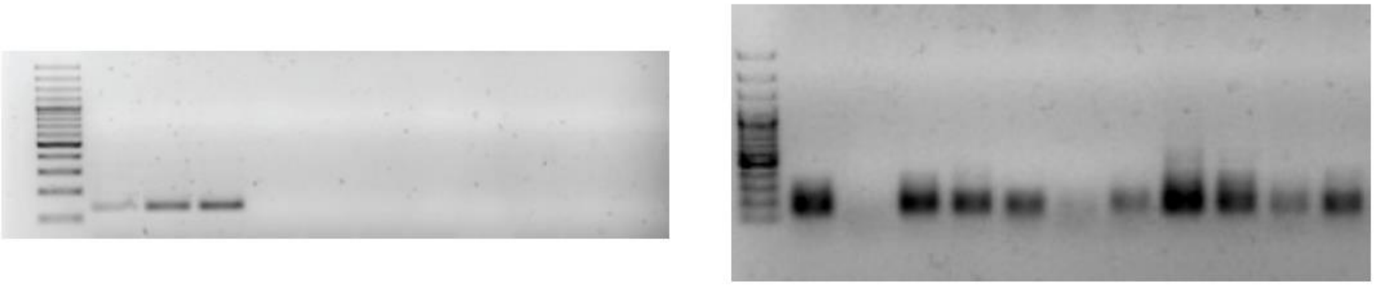

Figure S9. Original Agarose Gels.

Table S1. Details of feline lymphoma sample cohort.

WHO 2008 CLASSIFICATION	SEX	NEUTER STATUS	AGE	BREED	MITOTIC COUNT
ALCL	f	y	23	European Shorthair	2
EATL I	f	y	15	European Shorthair	82
EATL I	f	y	16	European Shorthair	91
EATL I	f	y	16	European Shorthair	68
EATL I	m	y	11	European Shorthair	94
EATL I	f	y	16	European Shorthair	120
EATL I	f	y	11	European Shorthair	17
EATL I	f	y	14	European Shorthair	13
EATL I	m	y	13	European Shorthair	57
EATL I	f	y	22	European Shorthair	80
EATL I	m	y	14	Unknown	50
EATL I	f	y	12	European Shorthair	32
EATL I	m	y	22	European Shorthair	24
EATL I	m	y	12	European Shorthair	1
EATL I	m	y	15	European Shorthair	1
EATL I	f	y	12	European Shorthair	70
EATL II	m	y	14	European Shorthair	1
EATL II	m	y	19	European Shorthair	10
EATL II	m	y	12	Brit. Longhair	0
EATL II	m	y	18	European Shorthair	0
EATL II	f	y	16	Brit. Longhair	2
EATL II	f	y	21	European Shorthair	7
EATL II	f	y	20	European Shorthair	0
EATL II	m	y	16	European Shorthair	1
EATL II	f	y	17	European Shorthair	0
EATL II	f	y	9	Unknown	1
EATL II	m	y	24	European Shorthair	0
EATL II	m	y	6	Brit. Longhair	0
EATL II	f	y	21	Unknown	0
EATL II	f	y	14	European Shorthair	0
EATL II	f	y	22	European Shorthair	0
EATL II	m	y	26	European Shorthair	1
EATL II	m	y	15	European Shorthair	0
PTCL	m	y	23	European Shorthair	3
PTCL	m	y	19	European Shorthair	113
PTCL	f	y	16	European Shorthair	1
PTCL	m	y	13	European Shorthair	121
PTCL	f	y	14	European Shorthair	108
PTCL	f	y	14	European Shorthair	87
PTCL	m	y	8	Maine Coon	117
PTCL	f	y	13	European Shorthair	123
T	f	y	8	European Shorthair	47

Table S2. Oligonucleotides for PCR, qPCR and ARMS-qPCR assays.

PCR

Primer name	Primer sequence (5' -> 3')	Length [bp]	Tm [°C]	Amplicon length [bp]
STAT3_fw	CCT TCC CAT ATC CGA TGA CA	20	64.6	
STAT3_rev	CCA ACG TGG AAA ATC AAC AA	20	63.3	255
STAT5B_fw	CCC CTG GCT TTG ATA AAT GA	20	63.6	
STAT5B_rev	CGC AAC AAA ACC AGA TCA GA	20	63.8	378
FeLV_U3_exo_fw	AAC AGC AGA AGT TTC AAG GCC	21	64.4	
FeLV_U3_exo_rev	TTA TAG CAG AAA GCG CGC G	19	66.2	131
FeLV_U3_exo/endo_fw	CAG TGG TGC CAT TTC ACA AG	20	64.2	
FeLV_U3_exo/endo_rev	AGC CTG GAG ACT GCT GGT AG	20	63.6	190

qPCR and ARMS-qPCR

Oligonucleotide name	Primer or probe sequence (5' -> 3')	Length [bp]	Tm [°C]	Amplicon length [bp]
qSTAT5B_fw-consensus	TGC CTT TTA CCA CCA GAG ACT TC	23	56.9	72
qSTAT5B_rev-consensus/ ARMS	AGA TAA GGT AAT TCA GGT CCC CTA AG	26	55.5	
qSTAT5B_fw-ARMS c. 1924A>C	GAG GAA AGG ATG TTT TcG CA	20	53.2	97
qSTAT5B Hydrolysis probe (FAM)	*FAM-* CAT CCG GTC *-ZEN-* CCT GGC TGA CCG *-IABkFQ*	21	64.7	
qSTAT3_fw-consensus	CAA GCA GCA GCT GAA CAA CA	20	56.4	125
qSTAT3_rev-consensus/ ARMS	ACG CCT CCT CCT TAG GAA TG	20	56.4	
qSTAT3_fw-ARMS c. 1919A>T	CCA GTC CGT GGA ACC tTT T	19	55.2	146
qSTAT3_fw-ARMS c. 1981G>T	ATC ATG GGC TAT AAG ATC AcG TA	23	53.1	88
qSTAT3 Hydrolysis probe (FAM)	*FAM-* CC AGC GGA G *-ZEN-* A CAC GAG GAT GTT G *-IABkFQ*	23	61.8	

Tm: melting temperature of NetPrimer software (www.permierbiosoft.com/netprimer/)
Small letter in sequence of ARMS primer: mismatch with both alleles of the bi-allelic SNP site artificially introduced to enhance sequence discrimination
FAM: 6-carboxyfluorescein; ZEN™ internal quencher, IABkFQ: Iowa Black™ fluorescent quencher

Supplementary Data: Template dsDNA fragments for ARMS qPCR (based on assembly Felis_catus_9.0).

STAT3 signal transducer and activator of transcription 3 [Felis catus (domestic cat)], Gene ID: 101095698, updated on 24-Jun-2020 (based on assembly Felis_catus_9.0)

> feline *STAT3* DNA (wild type) - chrE1:42942211 - 42942599

```
GAAAAATAAA TCAGGTAGTT TTCTCTAAGA TTACCTGGCC ATTATCCTCC  50
CTTCCCATAT CCGATGACAG GCAAGACCCA GATCCAGTCC GTGGAACCAT  100
ATACCAAGCA GCAGCTGAAC AACATGTCGT TTGCTGAAAT CATCATGGGC  150
TATAAGATCA TGGATGCTAC CAACATCCTC GTGTCTCCGC TGGTTTATCT  200
CTACCCGGAC ATTCCTAAGG AGGAGGCGTT TGGAAAGTAT TGTCGACCAG  250
AAAGCCAGGA GCATCCTGAA GCTGACCCAG GTAGTTGTTG ATTTTCCACG  300
TTGGGCACTT TTTCTGGGAA AAAATGGGAA ATTGCAGGAT TCTTGGAGGA  350
TAGGTAGGTC ACTGCCCAAA GAGCCTGGTG ACCTTTATT  389
```

> feline *STAT3* DNA (incl. SNPs)- chrE1:42942211 - 42942599

```
GAAAAATAAA TCAGGTAGTT TTCTCTAAGA TTACCTGGCC ATTATCCTCC  50
CTTCCCATAT CCGATGACAG GCAAGACCCA GATCCAGTCC GTGGAACCAT  100
tTACCAAGCA GCAGCTGAAC AACATGTCGT TTGCTGAAAT CATCATGGGC  150
TATAAGATCA TGtATGCTAC CAACATCCTC GTGTCTCCGC TGGTTTATCT  200
CTACCCGGAC ATTCCTAAGG AGGAGGCGTT TGGAAAGTAT TGTCGACCAG  250
AAAGCCAGGA GCATCCTGAA GCTGACCCAG GTAGTTGTTG ATTTTCCACG  300
TTGGGCACTT TTTCTGGGAA AAAATGGGAA ATTGCAGGAT TCTTGGAGGA  350
TAGGTAGGTC ACTGCCCAAA GAGCCTGGTG ACCTTTATT  389
```

STAT5B signal transducer and activator of transcription 5B [Felis catus (domestic cat)]; Gene ID: 101090663, updated on 12-Feb-2020

> feline *STAT5B* DNA (wild type) - chrE1:42844241 - 42844617

```
CTGTGGTTAA TTTTAAATGG AGATCTTTAT TGGGACAGTT TTAACGGAGA  50
GTCTTTAATT TGTAGCATGT TTGGATTTTT TAACATTTCC TGTTCAGAAA  100
TCATGTTTAG AATACGATTA TTCTGTTTGT CGCTCTAGAG GAAAGGATGT  150
TTTGGAATCT GATGCCTTTT ACCACCAGAG ACTTCTCCAT CCGGTCCCTG  200
GCTGACCGCT TAGGGGACCT GAATTACCTT ATCTACGTGT TTCCTGATCG  250
GCCAAAAGAT GAAGTGTACT CCAAATACTA CACACCAGTC CCATGCGAGC  300
CTGCCACTGG TAACAATGTT CGCATTCTGA TCTGGTTTTG TTGCGATTTG  350
AGTTTCTTT TGGTTTTTGT GTGGGTG  377
```

> feline *STAT5B* DNA (incl. SNP)- chrE1:42844241 - 42844617

```
CTGTGGTTAA TTTTAAATGG AGATCTTTAT TGGGACAGTT TTAACGGAGA  50
GTCTTTAATT TGTAGCATGT TTGGATTTTT TAACATTTCC TGTTCAGAAA  100
TCATGTTTAG AATACGATTA TTCTGTTTGT CGCTCTAGAG GAAAGGATGT  150
TTTGGcATCT GATGCCTTTT ACCACCAGAG ACTTCTCCAT CCGGTCCCTG  200
GCTGACCGCT TAGGGGACCT GAATTACCTT ATCTACGTGT TTCCTGATCG  250
GCCAAAAGAT GAAGTGTACT CCAAATACTA CACACCAGTC CCATGCGAGC  300
CTGCCACTGG TAACAATGTT CGCATTCTGA TCTGGTTTTG TTGCGATTTG  350
AGTTTCTTT TGGTTTTTGT GTGGGTG  377
```
